# A Novel Therapeutic Approach of 980 nm Photobiomodulation Delivered with Flattop Beam Profile in Management of Recurrent Aphthous Stomatitis in Paediatrics and Adolescents—A Case Series with 3-Month Follow-Up

**DOI:** 10.3390/jcm13072007

**Published:** 2024-03-29

**Authors:** Reem Hanna, Ioana Cristina Miron, Stefano Benedicenti

**Affiliations:** 1 Department of Surgical Sciences and Integrated Diagnostics, University of Genoa, 16132 Genoa, Italy; 5632651@studenti.unige.it (I.C.M.); stefano.benedicenti@unige.it (S.B.); 2 Department of Restorative Dental Sciences, UCL-Eastman Dental Institute, Medical School, University College London, London WC1E 6DE, UK; 3 Department of Oral Surgery, King’s College Hospital NHS Foundation Trust, Denmark Hill, London SE5 9RS, UK

**Keywords:** 980 nm, diode laser, free radicals, lesion resolution, LLLT, oxidative stress, pain intensity, PBM, photobiomodulation, recurrent aphthous stomatitis

## Abstract

**Background/Objectives**: Recurrent aphthous stomatitis (RAS) is one of the most common oral mucosal lesions and a very debilitating lesion, especially in paediatric and adolescent patients. The current pharmacotherapy offers a pain relief but not without side effects, and therefore photobiomodulation (PBM) can be an alternative therapy. To the authors’ best knowledge, no published study has explored the efficacy of λ 980 nm laser PBM in the management of all RAS subtypes in paediatric and adolescent patients, and therefore, this prospective observational clinical study was conducted to bridge this gap by evaluating λ 980 nm laser PBM efficacy in symptomatic RAS management in paediatric and adolescent patients. The objectives were to evaluate (1) pain intensity alleviation; (2) wound healing rate; (3) wound size closure; (4) a complete resolution; (5) evidence of recurrence; and (6) patients’ treatment satisfaction. **Methods**: The study’s variables were assessed at the following timepoints: T0: pre-treatment; T1: immediately after first PBM session; T2: 5 hours (h) post first PBM session (via telephone call); T3: immediately after second PBM session (three days post first PBM session); T4: three-day follow-up (after complete PBM treatments); T5: two-week follow-up; and T6: three-month follow-up. The following PBM dosimetry and treatment protocols were employed: λ 980 nm; 300 mW; 60 s; 18 J; CW; flattop beam profile of 1 cm^2^ spot size; 18 J/cm^2^; and twice-a-week irradiation (72 h interval). **Results**: At T1, significant immediate pain intensity relief was reported. 33.33% recorded “4” and 66.67% reported “5” on the quantitative numeric pain intensity scale (NPIS), and this continued to improve significantly (83.33%) at T2. All the subjects reported “0” on the NPIS at T3, T4, T5 and T6. There was a significant reduction in the lesion surface area (>50% complete healing) at T3 compared to T0. Complete healing (100%) with no evidence of scarring and lesion recurrence observed at T4, T5 and T6. Very good patients’ satisfaction was reported at all timepoints. **Conclusions**: This is the first report demonstrating λ980 nm efficacy in all RAS subtype management in paediatric and adolescent patients with a 3-month follow-up, whereby its PBM dosimetry and treatment protocols were effective from scientific and practical standpoints, and hence multicentre RCTs with large data are warranted to validate its reproducibility and to enrich the knowledge of PBM application in all RAS subtypes.

## 1. Introduction

Recurrent aphthous stomatitis (RAS) in paediatric and adolescent patients is usually a benign, idiopathic, chronic and recurrent inflammatory oral mucosal disorder and, in some cases, can represent a sign of a systematic disorder [1]. It remains one of the most common oral mucosal lesions in the paediatric and adolescent population as its prevalence among this group is 39% but ~20% in the adult population [2,3]. 

RAS aetiology remains unclear and not fully understood. Hence, obtaining a comprehensive medical and nutritional history from the patients or legal guardians supplemented with a thorough clinical examination by an experienced clinician is essential for a definitive diagnosis [4].

### 1.1. Clinical Presentation and Types of RAS

RAS is characterised in being painful and recurrent at various degrees of severity [5], with a great impact on patients’ functional activities such as mastication, swallowing and speech [6]. The clinical presentations of an aphthous ulcers are as follows: one or more in number, rounded in shape, superficial, tender by palpation and can remain between a few days to a few months. During its initial phase, a localised erythema develops. Subsequently, within hours, small white papules are formed, which become ulcerated and slowly enlarged over the following 48–72 hours (h) [7]. 

Moreover, RAS has a distinctive burning sensation that persists between 2 and 48 h prior to the appearance of the ulcer and can last between 7 and 14 days. It tends to reappear at intervals between a few days to a few months [8]. The clinical presentation of the RAS subtypes is as follows [1]: (1) minor aphthous ulcer; (2) major aphthous ulcer; and (3) herpetiform aphthous ulcer [8].

### 1.2. Environmental and Systematic Factors Contributing to RAS Aetiology

The literature reported many predisposing factors that contribute to RAS aetiology [9] such as local trauma [10]; genetic [11]; nutritional deficiencies [12]; oral microbiota derangements [13]; immune [14,15] and endocrine diseases [16]; medications [17]; stress [18]; or allergies [11]. 

### 1.3. RAS Pathogenesis

Although RAS pathogenesis remains poorly understood, it is most likely related to immune response modifications [19] and oxidant–antioxidant imbalances [20,21], favouring oxidative damage [22]. The mechanism of cell-mediated immune response plays an essential role in RAS pathogenesis by initiating the T cells and generating tumour necrosis factor-α (TNF-α) by leucocytes (macrophages and mast cells) [23]. Additionally, light and electron microscope examinations of oral aphthous ulcers showed an early penetration and infiltration of the lympho-monocyte.

The excessive or unopposed production of interluekine-6 (IL-6) or IL-1 plays an important role in the pathogenesis of oral aphthosis [24]. Moreover, the secretion of anti-inflammatory cytokines, transforming growth factor-β (TGF-β) and IL-10 was significantly decreased in patients with RAS, indicating an imbalance in pro- and anti-inflammatory cytokines production, and hence may contribute to the development of autoimmunisation and RAS [24].

As oxidant–antioxidant imbalance contributes to RAS pathogenesis; it triggers free radicals and can perturb energy homeostasis and inflammatory pathways [25], leading to oral mucosal ulceration. In physiological circumstances, cells have an antioxidant mechanism containing enzymes such as superoxide dismutase, catalase and GPx. Non-enzymatic antioxidants are vitamins A, E, C, melatonin, UA and reduced glutathione (GSH) [26]. However, the enzymatic antioxidant mechanism is impaired in patients with active RAS, and it appears to play an important role in the disease pathogenesis [27]. 

A study conducted by Tugrul et al., 2016 [22] showed that the total oxidative status and oxidative stress index values were significantly higher in patients with RAS compared to the control group, whereas the antioxidant status values were significantly lower. Additionally, it was observed that the DNA damage in the RAS group was significantly higher than the control group.

### 1.4. Current Treatment Modalities

#### 1.4.1. Pharmacotherapy

Since the main aetiology of RAS remains unclear, the treatment has been always to alleviate symptoms [21]. 

The current treatment modalities include topical analgesic and anaesthetic agents, corticosteroids, antibiotics, multivitamins, cauterisation and combined therapy [4,28]. Topical treatment aims to prevent infection, provide analgesic effects, reduce inflammation and treat active ulcers [3]. The most common topical treatments are local anaesthetics, antiseptics and topical steroids [4], whereas the systemic treatment is exceptional in paediatric patients and should be considered only in children with immunity deficiency [3]. One of those possible drugs is pentoxifylline, which inhibits TNF-α production and other pro-inflammatory cytokines [1]. This drug is, however, not indicated in children under the age of 18-year-old [29,30]. Moreover, the main systematic therapies can induce side-effects such as; steroid medications causing candidiasis [4], and oral rinses based on tetracycline or chlorhexidine content shortening the ulcer healing time, but are contraindicated in children, as they can induce tooth discoloration [4,31]. 

Systemic medication can be considered when the topical treatment is ineffective, but the Cochrane review showed that the effect of systematic medications does not outweigh the negative side effects [30], and hence the commonly accepted standard treatment modality for RAS remains to be poorly established [4].

#### 1.4.2. High-Level Laser Therapy (Surgical Laser)

High-level laser therapy (HLLT) of various wavelengths has been utilised in the management of several oral mucosal lesions [32,33,34]. A systematic review conducted by Sutter et al., 2017 [35] included a number of RCTs studies [36,37,38,39] that utilised HLLT of various surgical wavelengths (CO_2_, Nd:YAG, diodes) with a diverse range of laser parameters compared to the topical treatment in the management of RAS in adult patients. The results varied but showed a positive response in reducing pain intensity and wound healing time, but the level of evidence of those studies ranged between 0 and 2 according to the Jadad score. This score is a methodological quality of a clinical trial measured by objective criteria ranging from 0 to 5 in which a score ≥ 3 is considered to entail a high quality of evidence [40]. Hence, the authors of this review suggested further robust studies with large data and standardised assessment tools to ensure high levels of evidence. 

#### 1.4.3. Photobiomodulation Therapy

The scientific literature has reported the evidence of photobiomodulation (PBM) therapy in alleviating pain [41,42], promoting wound healing [43] and anti-inflammatory properties [44], which ultimately play a pivotal role in RAS management.

At cellular and molecular levels, PBM photons are absorbed by cytochrome c oxidase (photoacceptor) on the outer membrane of the mitochondria, resulting in cellular and molecular cascades, stimulating more adenosine triphosphate (ATP) production, inducing nitric oxide and low levels of reactive oxygen species (ROS), which subsequently activate the transcription factors such as NF-κB, inducing many gene transcript products responsible for PBM beneficial effects [45].

The analgesic effect of PBM, however, is primarily due to an increase in the release of β-endorphin, serotonin and enkephalins (natural endogenous opioid neuropeptides), acting to attenuate substance P release and bradykinin, histamine and prostaglandin E2 secretions, leading to an inhibition in the pain afferent fibres [46], leading to reversible changes in the membrane permeability; therefore, the therapeutically applied photons stimulate cell activity and proliferation and reduce in the activity of the C and A delta fibres [47,48]. 

Moreover, PBM alters nerve conduction and excitation in the peripheral neurons by its action on Na+/K+ pump channel, resulting in noxious stimuli reduction, through its effects on transient receptor potential cation channel subfamily V member 1 (TRPV1) and nerve growth factor (NGF) signalling blockers, decreasing their expressions (blockage of inflammatory thermal hyperalgesia) [41]. 

At supra-cellular level, PBM has been correlated with an increase in microcirculation, modulating neurotransmissions and improving nerve regeneration, an increase in fibroblasts and macrophages proliferation [45] and inhibiting inflammatory cytokines, demonstrated by upregulating TNF-α, IL-1β, IL-6 and IL-8 levels and elevating intracellular levels of cAMP [44]. Taking this into account, it is noteworthy that PBM can be a significant tool in RAS management; a study conducted by Xiao et al., 2023 [49] showed that a panel of cytokines such as IL-6, TNF-α and IL-2 represent significant indicators for patients with RAS.

### 1.5. Rationale in Conducting the Present Study

The scientific literature is very scarce in utilising PBM in RAS management. A recent systematic review conducted by Khaleel et al., 2020 [50] concluded that PBM laser therapy was better in treating aphthous ulcer lesions compared with topical medications, and all laser wavelengths in the included report were shown to be effective. However, the results should be interpreted with caution as none of the included studies demonstrated a low risk of bias in all the assessed domains.

Another recent systematic review conducted by dos Santos et al., 2020 [51] aimed to evaluate PBM efficacy in the management of RAS in an adult cohort. It highlighted methodological limitations in their included studies and presented with a moderate or a high risk of bias. These methodological flaws indicated an inadequate literature search strategy, the absence of a pre-defined review protocol and the lack of an explanation of the selection design for inclusion. A high degree of heterogeneity in relation to the study design, laser therapy parameters, protocols of administration and control group intervention was well-noticed, and due to a small number of the included studies, this made a meta-analysis infeasible. Moreover, a discrepancy in the PBM parameters applied by the selected RCTs in this review made it impossible for the authors to extrapolate a specific protocol for future clinical studies.

All the clinical studies that have been established up to date were related to utilising PBM in the management of RAS in adult patients aged ≥18-years-old, except one RCT study conducted by Bardellini et al., 2020 [52], which utilised λ 645 nm in only minor RAS (MiRAS) management in children with a mean age of 8.9 ± 2.2 and concluded no statistical significance observed between the control (sham) and the PBM groups in terms of a pain alleviation timeframe and wound size reduction (healing time).

Additionally, to the best knowledge of the authors, there is no published study that has explored the efficacy of λ 980 nm PBM in the management of any other types of RAS in paediatric and adolescent patients, and therefore, our study was conducted to bridge this scientific literature gap, improve the clinical understanding and underpin PBM efficacy in RAS management in paediatric and adolescent patients as a standardised therapy. Hence, the aim of the present study was aimed to evaluate λ 980 nm laser PBM efficacy in the treatment of symptomatic RAS in paediatric and adolescent patients. The objectives were as follows: to alleviate pain intensity and the total healing time of aphthous ulceration. The following objectives were to evaluate (1) pain intensity alleviation (at rest and during functional activities); (2) wound healing rate (timeframe); (3) wound size closure; (4) complete resolution; (5) evidence of recurrence; and (6) patients’ treatment satisfaction. So, our null hypothesis was λ 980 nm laser PBM has no significant effect in the treatment of symptomatic RAS.

## 2. Materials and Methods

### 2.1. Study Design

A prospective observational clinical study of six subjects aged <18 years old presented with oral RAS at an onset of <24 h. A λ 980 nm PBM laser (Doctor Smile, Lambda, Vincenza, Italy) was employed, and its photonic energy was delivered with a flattop beam profile hand-piece (Doctor Smile, Lambda, Vincenza, Italy). 

The primary and secondary outcomes in terms of a complete resolution of the lesion, pain alleviation at rest and during functional activities, evidence of lesion recurrence, erythema and exudate manifestation and patient’s treatment satisfaction were evaluated at the following timepoints and follow-up period: T0: pre-treatment (at clinic); T1: immediately after 1st PBM session (at clinic); T2: 5 h post 1st PBM session (via telephone call); T3: immediately after 2nd PBM session (i.e., three days post 1st PBM session); T4: three-day follow-up (after complete PBM sessions) (at clinic); T5: two-week follow-up (at clinic); and T6: three-month follow-up (at clinic). This was based on a three-calibrated protocol at the clinic.

The investigator is an experienced clinician who performed the treatment for all the cases and assessed the outcomes with two independent experienced clinicians, who were not involved in the study, at T0, T1,T3, T4, T5 and T6 (at clinic). Additional roles for those independent clinicians were to collect and analyse the data and store them securely on an Excel spreadsheet. 

At T2 timepoint, an independent experienced assessor nurse conducted the follow-up appointment via a telephone call to collect the following data: pain intensity score; erythema scoring; and patients’ treatment satisfaction. They also had to analyse the data and store them on an Excel spreadsheet. This calibration process was employed to minimise the interobserver variability and bias. 

Different treatment options were discussed with the patients’ parents and legal guardians, explaining the pros and cons of each treatment, and all the patients opted to have the photobiomodulation treatment.

The study was conducted in accordance with the Declaration of Helsinki. Informed written consent was obtained from all the patients’ parents and legal guardians, and a full explanation of the treatment was provided, including a patient information leaflet. Additionally, informed written consent was obtained in relation to publishing the patients’ clinical photos and the study in a scientific peer-reviewed journal.

### 2.2. Interventional Group

The eligible subjects were fit and healthy, of both genders, aged <18, presented with one or more lesions of any oral RAS subtype at an onset < 24 h at the 1st PBM session and had never received PBM therapy. Within the inclusion criteria, parents and legal guardians of the recruited subjects demonstrated an understanding of the study and a willingness to participate and provide informed written consent.

The diagnosis of RAS was based on the patients’ anamnesis and clinical symptoms conducted thorough clinical examinations (extra and intraoral). Despite the fact that there is no specific diagnostic test for RAS, it is essential to exclude any possible underlying systemic causes [53]. Hence, all the recruited subjects were screened, and their general medical practitioners were consulted and confirmed their clear medical history. Hence, subjects with the following criteria were excluded: (1) subjects with a known systemic disease predisposing them to RAS (e.g., Behçet disease); (2) subjects undergoing systemic treatment for RAS; (3) the presence of a serious medical condition; (4) subjects who were treated with topical or systemic corticosteroid or antibiotics or analgesics a month prior to their enrolment in the study; and (5) subject with viral herpetic lesions.

### 2.3. Study’s Focused Question and PICO

The study’s focused question was Does λ 980 nm laser PBM alleviate pain intensity and accelerate oral wound healing in patients with all RAS subtypes? And then, the PICO score (P: Population; I: Intervention; C: Comparison; O: Outcomes) was formulated as follows:

P: Subjects who were <18 years old, presented with any subtype of oral RAU, which occurred no more than 48 h prior to their first PBM session, and diagnosed, with a detailed clinical history and examination of the ulcers and systemic tests [53].
I: λ 980 nm laser PBM irradiation
C: Not applicable.
O: Pain intensity (at rest and during functional activities), wound healing rate, complete lesion resolution, evidence of lesion recurrence, patient treatment satisfaction and parent’s positive experience. All these outcomes were based on qualitative and quantitative measures.

### 2.4. Therapeutic Photobiomodulation Protocol

To improve the standardisation and repeatability of PBM therapy, a λ 980 nm laser device was employed and delivered with a flattop beam profile hand-piece. λ 980 nm was chosen due to its deep penetration depth properties [54], and also the flattop beam profile delivery system offers a uniform distribution of the laser photonic over 1 cm^2^ of a target surface area [55,56]. The therapeutic output power was measured by the Pronto-250 power meter (Gentec Electro-Optics, Inc., Quebec, QC, Canada). Table 1 illustrates the laser device specifications, PBM dosimetry and treatment protocols. 

### 2.5. Description of λ 980 nm Irradiation with Flattop Beam Profile Application

The investigator is an experienced clinician and who purchased the laser device and the flattop delivery system more than six years ago and regularly utilises them in their daily practice. All the laser safety measures were respected in accordance with the American National Standards Institute (ANSI) guideline [57]. 

**Figure 1:**
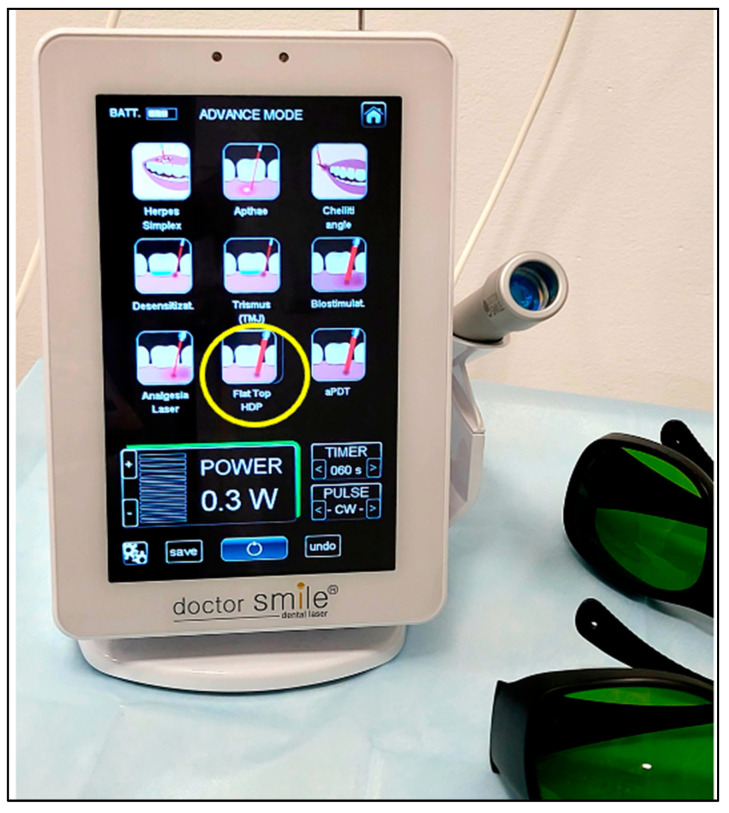
The laser device of λ 980 nm where the yellow circle indicates PBM therapy with a delivery system of flattop beam profile, and the panel also shows the therapeutic power output “0.3 W”, irradiation time “60 seconds (s)” and emission mode “continuous emission mode (CW)” which were utilised in the study. Eyewear protection (Green in colour) was worn by the operator, patient, nurse and patient’s parent.

The laser PBM dosimetry was as follows (Table 1): λ 980 nm PBM laser; therapeutic power output: 0.3 W (300 mW); irradiation time: 60 s; emission mode: CW; and beam profile: flattop (Figure 1). The device was calibrated and checked prior to the treatment.

**Figure 2:**
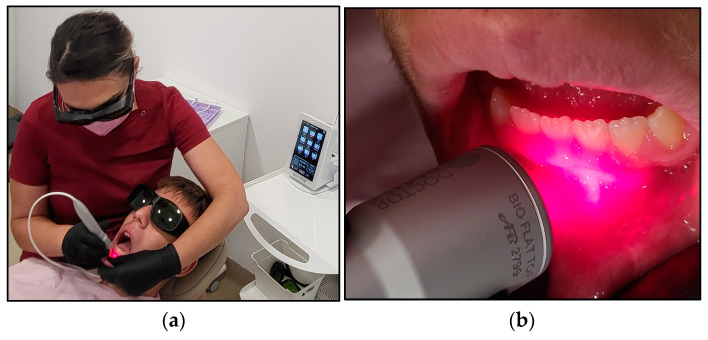
The irradiation process in case #2. (**a**) shows the operator performing PBM irradiation aphthous lesion on case #2, using the flattop beam profile to deliver the photonic energy of λ 980 nm via a static technique, and the patient appears very comfortable and cooperative during the PBM treatment. (**b**) illustrates the direction of the flattop beam profile at few millimetres distance from the target tissue and perpendicular to the target tissue.

The PBM irradiation process and technique are illustrated in Figure 2. The operator identified the area where the RAS was present. Two independent assessors and the operator recorded the following data prior to the PBM treatment at T0: pain intensity at rest and during functional activities, lesion size measurements, erythema status and patients’ treatment cooperation and satisfaction as well as the parents’ positive experience. All these data were stored on Microsoft Excel by the independent assessors. Then, the operator retracted the mobile soft tissue around the lesion in order to isolate the lesion and then placed the flattop delivery system about 2 mm from the lesion (non-contact) in a perpendicular direction. The lesion was irradiated for 60 s via a static application technique.

The following data were recorded at T1 (immediately after the 1st PBM session): pain intensity at rest and during functional activities; patient cooperation; patients’ treatment satisfaction including their parents’ positive experience.

### 2.6. Study’s Outcomes

The primary endpoint was a complete resolution of the aphthae ulceration, whereas the secondary endpoints were as follows: pain intensity alleviation at rest and during functional activities; evidence of no lesion recurrence; no erythema and exudate manifestation; patient’s treatment satisfaction; parents’ positive experience. 

### 2.7. Outcome Assessment Measures

#### 2.7.1. Visual Analogue Scale

**Figure 3:**
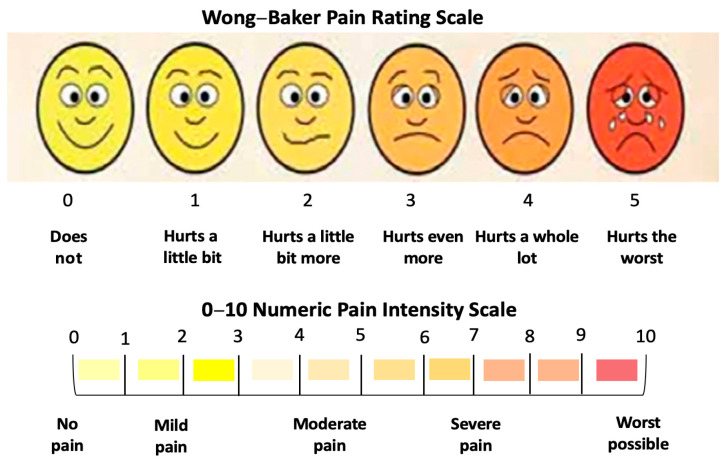
Wong–Baker Pain Rating Scale.

The patients’ reported data on pain intensity were evaluated using the visual analogue scale (VAS), which is the gold standard assessment tool for pain intensity [58]. The Wong–Baker Faces Scoring Scale was utilised. It corresponds to a quantitative numeric pain intensity scale (NPIS), ranging from “0”—does not hurt to “5”—hurts the worst. This corresponds to a numerical pain intensity scale (NPIS) (0–10), whereby a value of “0” indicates “no pain” and “10” represents the “worst possible” pain (Figure 3). 

A prospective clinical study conducted by Hanna et al., 2018 demonstrated that this assessment tool is reliable, reproducible, effective and suitable for paediatric and adolescent patients [33]. The patients, parents and legal guardians were familiarised with this assessment tool at the consultation appointment and were provided with a physical-coloured template of this tool [33] to be utilised during the T2 timepoint. The patients’ self-reported pain intensity was recorded at the following timepoints: T0, T1, T2, T3, T4, T5 and T6 and at rest and during functional activities: speaking, eating, smiling and brushing teeth.

#### 2.7.2. Clinician Erythema Assessment (CEA) Visual Assessment Scale 

The degree of the lesion erythematic area was evaluated by the main investigator based on a four-point scale, ranging from 0: none to 4: severe erythema with fiery redness (Table 2) [59]. 

The patients’ parents and legal guardians were familiarised with this assessment tool at the consultation appointment and were provided with a written template of this tool. This variable was recorded at the following timepoints: T0, T2, T3, T4, T5 and T6.

#### 2.7.3. Ruler Method for Wound Surface Area Measurement

**Figure 4:**
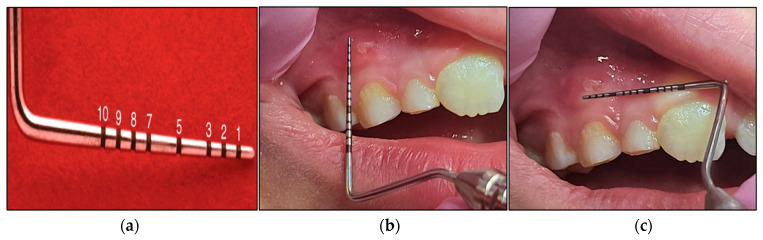
The method of measuring the wound surface area with periodontal probe in case #5. (**a**) A periodontal probe with black tick marks in which each one represents 1 mm, and hence the labled numbers represent the sequence of the measurement; (**b**) shows the length measurement of the wound (1.8 mm); (**c**) shows the periodontal in place measuring the width of the wound (2.5 mm), and hence, the wound surface area was 4.5 mm^2^.

The most common tool to determine wound surface areas is the ruler technique [60]. We utilised a periodontal probe with black tick marks, in which 1 mm (mm) represents one black tick mark (Figure 4a), allowing us to measure the width and length of the lesion as it is shown in Figure 4b,c (case #6), respectively. 

The wound surface area can be approximated and measured by multiplying the greatest length by the perpendicular width measurements and valued in millimetre (mm^2^) [61]. The scores for the clinical improvement were evaluated based on a “higher score” indicating “worst outcome” and the “lowest value” indicating “the best outcome”. This technique is simple, quick and affordable [62].

The measurements were performed by the investigator and another two independent clinicians who were not involved in the study. The mean value of the three measurements was obtained and stored on Microsoft Excel by the independent assessors and recorded at T0, T3, T4, T5 and T6 to evaluate the lesion healing rate (wound closure) and the lesion complete resolution rate.

#### 2.7.4. Wound Healing Grading Tool

The clinical grading of wound healing assessment was based on a four-point scale [63,64]: Grade I: represents a total healing; Grade II: moderate healing (>50% of RAS epithelialised and healed); Grade III: mild healing (<50% of RAS epithelialised and healed); Grade IV: represents no healing. This assessment was employed to record the wound-healing progress at T0, T3, T4, T5 and T6 timepoints.

#### 2.7.5. Patient Cooperation Assessment

The Frankl Behaviour Rating Scale (FBRS) was used to assess the participants’ level of cooperation during PBM treatment based on 4-levels as follows: 1: definitely negative; 2: negative; 3: positive; and 4: definitely positive [65]. Patients with level 1 of cooperation were excluded from the study. This was evaluated at T1 and T3 timepoints.

#### 2.7.6. Patient Treatment Satisfaction

**Figure 5:**
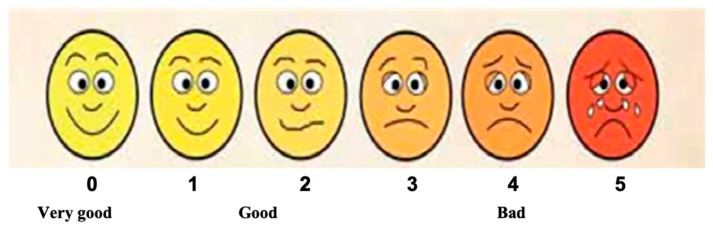
Modified Wong Baker Faces Rating Scale employed to evaluate patients’ treatment satisfaction, ranging from “0”, meaning very good, to “5”, meaning bad (adapted from Hanna et al., 2016, permission obtained [33]).

A modified Wong–Baker Faces Rating Scale (MWBFRS) was employed [33] to evaluate patients’ treatment satisfaction. It is based on the patient’s self-reported scoring, ranging from “0”: very good to “4–5”: bad (Figure 5). 

A prospective clinical study conducted by Hanna et al., 2018 [33] demonstrated that this assessment tool is reliable, effective and suitable for paediatric and adolescent patients. The patients, parents and legal guardians were familiarised with this assessment tool at the consultation appointment and were provided with a physical-coloured template of this tool [33]. This variable was recorded at the following timepoints: T0, T1, T2, T3, T4, T5 and T6. 

### 2.8. Statistical Analysis

The percentage is a statistical tool employed to express the relative amounts of increase or decrease in a standardised ratio comparison. It is a descriptive analysis of relatively simple calculations, providing a basic profile of what the data look like overall and showing proportions. The change in pain relief outcome values (quantitative variables) recorded at T0 and at different timepoints (T1, T2, T3, T4, T5 and T6) were expressed as percentages (%), as well as for the wound healing rate and resolution at T0 and at different timepoints (T3, T4, T5 and T6).

The statistical test for this calculation is called the z-test (one-sided) for the equality of two percentages using independent samples. A 5% level of significance is commonly utilised in statistics as it provides a balance between being too conservative and too liberal in accepting or rejecting a null hypothesis. 

The mean is a statistical tool that is calculated by adding the values in the dataset together and then dividing this by the number of added values.

## 3. Results

A concise and precise description of our experimental results and their interpretations are enlisted below.

### 3.1. Demographic Characteristics and Lesion Description

Six Caucasian subjects of both genders (three males and three females) with mean age of 9.33-year-old (ranged between 6 and 13) presented with various RAS subtypes at an onset which ranged between ~6 and 24 h. The total number of RAS lesions for the six subjects was 14 distributed in keratinised and non-keratinised buccal and labial mucosa (Table 3). 

The size, number, location and healing time are all dependent upon the subtype of RAS [52]. The total number of lesions in all the subjects was 14, in which 7 of them were the clustered small herpetiform type; 4 out of 14 lesions were MiRAS, and the remaining three were MaRAS.

Case #1 had one major RAS (MaRAS) and seven of the cluster herpetiform type of RAS, which were small in size. Only 10% of RAS classify as herpetiform in children (very rare) [66]. The lesions were very painful at rest, and the patient had difficulty in performing masticatory and phonatory functions. Any activity that involved the movement of the perioral zone led to an increase the pain intensity. Case #4 had one MaRAS and one MiRAS, whereas case #6 had one MaRAS, which is normally large, solitary, and can take a longer to heal than MiRAS [67], but this was not the case in our study. The remaining three cases (#2, #3 and #5) had a single MiRAS (50%), and this is in agreement with the literature as being the most common RAS (85%) [66] (Table 3). 

All the subjects’ symptoms were based on severe pain intensity at rest and during masticatory and phonatory functional activities (speaking, eating, brushing teeth and lip and cheek movements), and none of them presented with systematic symptoms throughout the treatment timepoints and follow-up period.

**Figure 6:**
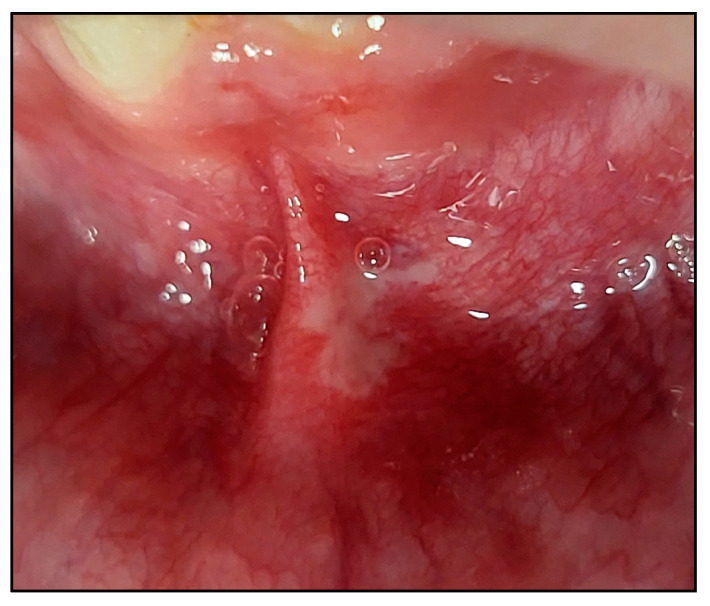
A clinical photo of a major RAS (MaRAS) (case #6) occupies the non-keratinised part of the labial mucosa, extending to the labial sulcus and spreading over the lateral border of the lower midline frenum. It was a white pseudomembranous with an erythematous halo surrounding the ulcer.

RAS subtype lesions presented in different clinical manifestations either as a well-circumscribed or undefined-shaped ulcer or with irregular edges, often depressed with an epithelial defect covered by a yellow–white pseudomembrane with an erythematous “halo” surrounding the ulcer [68] (Figure 6).

### 3.2. Primary Endpoints

#### 3.2.1. Pain Intensity Scoring at Rest

At T0, 50% of the subjects reported pain “8” on the NPIS, whereas 33.33% reported a score of “9” on the NPIS, and the remaining subject reported “7”. 

**Figure 7:**
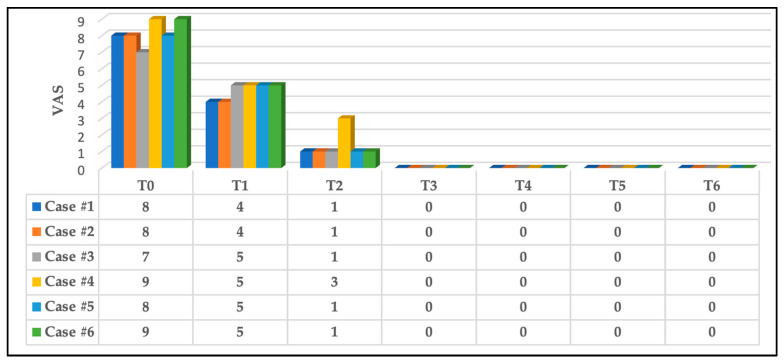
The results of pain intensity reported by all the subjects at rest. A significant improvement in pain reduction at T1, and this continued to be significantly reduced at T2, and all the subjects were pain-free at T3. This was maintained at T4, T5 and T6. Abbreviations: VAS: visual analogue scale; T0: pre-treatment (at clinic); T1: immediately after first PBM session (at clinic); T2: 5 h post first PBM session (via telephone call); T3: immediately after second PBM session at clinic (three days post first PBM session); T4: three-day follow-up (after complete PBM treatments, at clinic); T5: two-week follow-up (at clinic); and T6: three-month follow-up (at clinic).

At T1, significant immediate pain intensity relief was reported; 33.33% recorded “4” on the NPIS, whereas the remaining 66.67% reported “5”, and this continued to significantly improved 5 h after the first PBM session (T2) in which 83.33% (five out of six subjects) reported “1” on the NPIS, and the remaining subject (case #4) reported “3”. All the subjects reported “0” on the NPIS at T3, T4, T5 and T6. Figure 7 illustrates the results.

#### 3.2.2. Pain Intensity Scoring at Functional Activities

A reduction in pain intensity was reported in all patients during phonation, and muscular activities were reported at T1 (immediately after the first PBM session) compared to T0.

At T2 (5 h after the first PBM session), a significant pain reduction was reported, where 66.66% scored “2” on the NPIS, 16.16% (case #1) scored “3” and 16.16% scored “5” (case #4) on NPIS. This continued to improve at T3 (the second PBM session), where 83.33% (five out of six) scored “0”—no pain, and 16.66% scored “2” (case #4) on the NPIS.

At T4, the same five cases remained pain-free, but case #4 scored very mild pain (“1”) on the NPIS which was much less than in T3. This was related to a lack of the patient’s compliance with oral hygiene, and the patient was the youngest subject (6 years old) in the study’s cohort.

**Figure 8:**
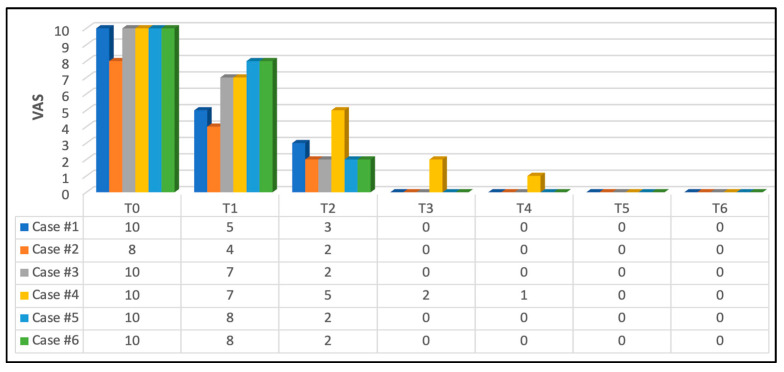
Patients’ pain self-reporting scores during phonation and muscular activities. Significant pain reduction at T1 (immediately after the first PBM session) compared to T0. This continued to demonstrate a substantial improvement in pain reduction at T2 (5 h after the first PBM session) where 66.66% scored “2” on NPIS, 16.16% (case #1) scored “3” and 16.16% scored “5” (case #4). This continued to improve at T3 (second PBM session) where 83.33% (five out of six subjects) scored “0” —no pain and 16.66% scored “2” on NPIS (case #4). At T4 (three-day follow-up post complete PBM treatments), the same five cases remained pain-free, but case #4 scored mild pain “1” on NPIS, which was much less than in T3. This was related to a lack of patient’s compliance with oral hygiene instructions. At T5 and T6, none of the subjects had pain, and all of them scored “0” on NPIS.

At T5 and T6, none of the subjects had pain; all scored “0”. Figure 8 illustrates the subjects self-reporting scores during functional activities (speaking, eating, brushing teeth and lip and cheek movements) at T0, T1, T2, T3, T4, T5 and T6.

### 3.3. Secondary Endpoints

#### 3.3.1. Effects on Erythema

In terms of erythema, at T0, three out of the six subjects (50%) had Grade 2 of erythema (mild erythema; definite redness); two out of the six subjects (33.33%) had Grade 3 of erythema (moderate erythema; marked redness) on the CEA visual assessment scale; and the remaining subject had Grade 4 (severe erythema; fiery redness) (16.66%). 

**Figure 9:**
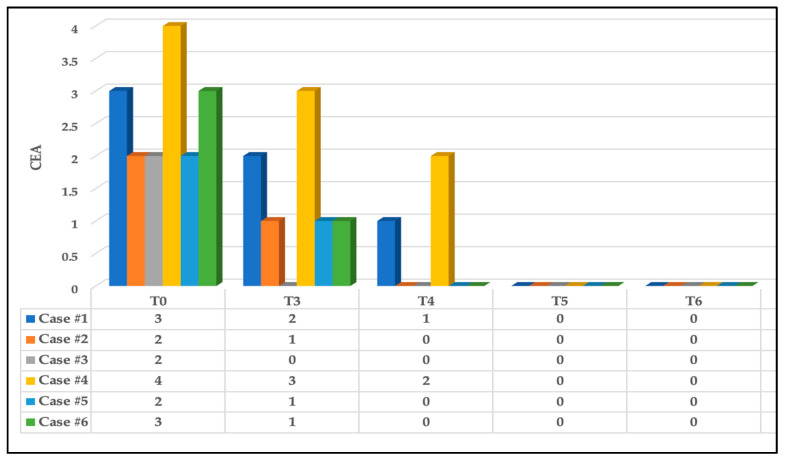
The clinical assessment scoring of erythema status of the six cases based on CEA visual assessment scale at different timepoints. Significant clinical improvement of the erythema reduction at T3 and T4 compared to T0 and continued to improve at T5 and T6 where no evidence of erythema noted.

At T3, there was a great improvement in the wound status in which 50% of the subjects had Grade 1 of erythema (almost clear; slight redness), 16.66% had Grade 2 (case #1) and 16.66% had Grade 0 (case #3) on the CEA visual assessment scale, whereas case #4 had Grade 3 (16.66%). This substantially improved at T4 (three-day follow-up after complete PBM treatment) in which 66.66% of the subjects had no erythema (Grade 0) and 16.66% had Grade 1 (case #1), whereas case #4 had Grade 2 (16.66%), which was related to subject #4’s lack of compliance with oral hygiene instructions. Nevertheless, at T5 and T6, there were significant improvements whereby all the cases had no erythema (Grade 0). Figure 9 illustrates the above findings.

#### 3.3.2. Wound Healing Rate

There was a significant reduction in the size of the lesion surface area at T3 compared to T0. In case #1, the MaRAS reduced to 2 mm^2^ compared to 5.6 mm^2^ at T0 (>50% complete healing, Grade II), whereas the six herpetiform lesions showed complete healing (Grade I, 100%), and the seventh lesion was reduced to 0.5 mm^2^ from 3.3 mm^2^ at T0 (>50% complete healing, Grade II). At T4, T5 and T6, there was complete healing (100% Grade I) with no evidence of recurrence.

Case #2, case #3 and case #5 (MiRAS) showed a significant reduction in the size of the wound surface area at T3, indicating >50% complete healing (Grade II) and complete healing at T4, T5 and T6, with no evidence of lesion recurrence. Whereas case #4 (MiRAS and MaRAS) showed a significant reduction in the size of the wound surface area (>50% complete healing) for both types of RAS, complete healing at T4, T5 and T6 was observed in MiRAS and at T5 and T6 in MaRAS. This could be contributed to case #4 reluctancy to comply with the oral hygiene instructions, and young age (6-year-old). 

Case #6 (MaRAS) showed a significant improvement in the wound healing at T3 where the wound surface area was reduced from 15 mm^2^ at T0 to complete healing at T3 (Grade I) and continued to maintain a remarkable healthy tissue with no evidence of recurrence at T4, T5 an T6.

Table 4 illustrates the above findings. Moreover, the wounds of all the subjects regardless of RAS subtypes healed with no scarring.

It is noteworthy that five out of the six cases showed a significant complete lesion resolution and closure at T3, and all the subjects showed 100% complete lesion resolution at T5 which was maintained at T6 without evidence of recurrence. 

**Figure 10:**
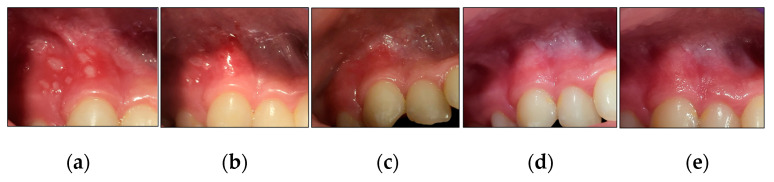
Clinical photos of case #1 illustrating the healing progression of one MaRAS and seven clusters of herpetiform RAS at different timepoints. (**a**) T0 (pre-treatment): one MaRAS and seven herpetiform lesions occupying the keratinised and non-keratinised buccal mucosa of UR3 and UR4, extending to buccal vestibule; (**b**) shows >50% complete healing (Grade II) at T3, whereas the seven herpetiform lesions showed complete healing (Grade I), apart from one lesion which showed >50% complete healing (Grade II); (**c**) shows all the lesions healed completely at T4, (**d**) shows two-week follow-up (T5), where all the lesions healed completely without evidence of erythema or recurrence; (**e**) shows 3-month follow period (T6) where no evidence of recurrence.

**Figure 11:**
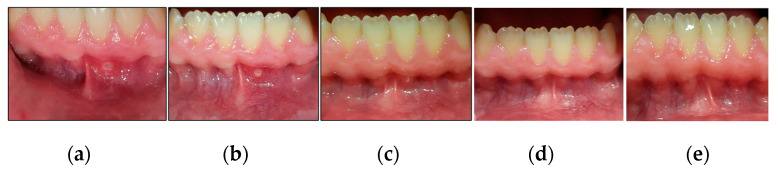
Clinical photos of case #2 illustrating the healing progression of MiRAS lesion at different timepoints and follow-up period. (**a**) shows an MaRAS lesion occupying the keratinised labial mucosa of LL1 at T0 (pre-treatment); (**b**) shows >50% complete healing of the lesion (Grade II) at T3; (**c**) shows the lesion healed completely (Grade I) at T4; (**d**) shows two-week follow-up (T5) with no evidence of recurrence; (**e**) shows 3-month follow-up period (T6) with no evidence of recurrence.

**Figure 12:**
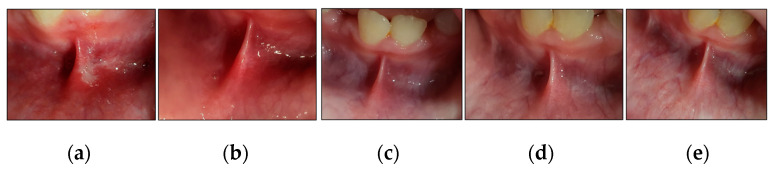
Clinical photos of case #6 illustrating the healing progression of MaRAS lesion at different timepoints and follow-up period. (**a**) shows an MiRAS lesion occupying the non-keratinised labial mucosa, extending to the labial sulcus and spreading over the lower midline frenum. It was a white pseudomembranous with an erythematous halo surrounding the ulcer at T0 (pre-treatment); (**b**) shows complete healing (Grade I) with no evidence of scarring at T3; (**c**–**e**) show the treated site maintained to be healthy with no evidence of lesion recurrence at T4, T5 and T6, respectively.

We outlined below the clinical outcomes of each type of RAS healing process of the present study; case #1 (MaRAS and herpetiform), case #2 (MiRAS) and case #6 (MaRAS) at T3, T4, T5 and T6 compared to T0 (Figure 10, Figure 11 and Figure 12).

#### 3.3.3. Patient’s Cooperation

The results showed that all the subjects were very cooperative at T1 and T3, and the score was “4” (very positive) on FBRS at those timepoints.

#### 3.3.4. Patient’s Treatment Satisfaction and Parent’s Positive Experience

All the patients were satisfied with the PBM treatments and scored “1” (good) on the MWBFRS, and equally, the parents expressed their positive experience as their children felt a significant immediate pain relief and rapid wound healing, which significantly helped the subjects during functional and phonatory activities.

## 4. Discussion

RAS is a debilitating oral mucosal lesion with a great impact on functional activities, especially in the paediatric and adolescent cohort. The lesion is characteristically observed in childhood and adolescence, with a frequent tendency to reoccure and is clinically presented in three forms: herpetiform RAS, MiRAS and MaRAS. Our study, for the first time, utilised PBM in the management of three RAS subtypes. 

A λ 980 nm PBM laser delivered with a flattop hand-piece showed to be effective in offering an immediate pain relief and rapid complete ulcer resolution with no evidence of lesion recurrence at the two-week (T5) and three-month (T6) follow-up timepoints. Additionally, all the subjects reported “very good” treatment satisfaction, and their parents/legal guardians reported a “positive experience”. 

### 4.1. Evaluation Pain Alleviation

#### 4.1.1. Evaluation of Pain Alleviation at Rest

The effects of PBM on pain relief onset varied among the scientific literature. Pain relief was differentiated between an immediate effect after treatment and relief in the days following laser use. 

Immediate pain relief was studied by three research groups treating RAS with non-thermal CO_2_ [34,39,69]. A study conducted by Sattayut et al., 2023 [69] showed no initial effect of CO_2_ on pain relief, but on the third day, pain alleviation was reported. This is in disagreement with other two studies conducted by Pradad et al., 2013 [34] and Zand et al., 2009 [39] which demonstrated an immediate pain relief at 24 h post CO_2_ irradiation. This could be contributed to a lower employed output power setting. 

In the present study, an immediate effect of a λ 980 nm PBM laser on pain relief was reported at T1 (immediately after the first PBM session), where the pain score was significantly reduced from the mean score of all the subjects which was at a value of “8.16” at T0 (pre-treatment) to a value of “5.66” at T1 (50% reduction in pain) on the NPIS. Moreover, the subjects reported to have further significant pain relief at T2 (5 h after the first PBM session), where the mean score value was “1.33” on the NPIS, indicating a significant reduction of 7-fold in the pain score on the NPIS. Remarkably, at T3 (immediately after the second PBM session—72 h after the first PBM session), all the subjects reported no pain and scored “0” on the NPIS, and this was maintained at T4, T5 and T6. This signifies that PBM laser therapy is not only effective in relieving pain but has an immediate effect compared with high-level laser therapy (surgical laser) [34,39,69]. This is a very important pillar in the management of RAS in children and adolescents.

A recent study conducted by Bardellini et al., 2020 [52] showed a significant pain reduction on the fourth day after λ 645 nm PBM laser irradiation of RAS in a child cohort, and no pain was reported 10 days after the beginning of the treatment, whereas, in our study, the subjects reported no pain (100% pain relief) immediately after the second PBM session—72 h from the beginning of the PBM treatment. This might be contributed to the deeper penetration depth of λ 980 nm laser PBM [54] compared with λ 645 nm, targeting the inflammatory peripheral nerve ending, as well as employing a flattop delivery system that can offer a uniform distribution of the energy over 1 cm^2^ surface area of the target. This ultimately can contribute significantly in optimising an immediate analgesic effect. Additionally, we also considered the other PBM dosimetry in achieving optimal outcomes in our study [45] such as the minimal therapeutic output power (300 mW), irradiation time (60 s) and treatment protocol based on only two sessions per week with a 72-h time interval. Our chosen protocol is in agreement with a study conducted by Enwemeka et al., 2004 [70] which showed the effectiveness of low-power lasers (<500 mW) on sores and ulcer wounds and concluded that laser therapy is effective at repairing tissue and controlling pain, although the outcomes may be influenced by the wavelength of the laser. This has been well documented by several studies revealing that the PBM laser operating parameters at infrared wavelengths led to more effective pain reduction [41,42,43,48]. Hence, PBM therapy, in the present study, was effective in offering 50% pain intensity alleviation immediately after the first PBM session, which is very crucial in children and adolescents. This ultimately offered a positive patient’s experience and “very good” treatment satisfaction, and this also applied to the parents’ satisfactory experience [52]. 

A study conducted by Hussein et al., 2021 utilised λ 980 nm in treating RAS in an adult cohort and reported a statistically significant decrease in pain on the second day, and further pain reduction was observed on the seventh day [71]. This was in agreement with another study conducted by Farist et al., 2014 which utilised the same wavelength, λ 980 nm, as the latter study at a power output of 1 W in non-contact mode, and pain reduction was reported on the third and seventh day of the PBM treatment [72]. This coincided with another study conducted by Dhopte et al., 2022 utilising another near infrared wavelength, λ 810 nm, and pain reduction was reported on the third day of the PBM treatment of RAS in adults [73]. However, our study demonstrated a significant pain reduction reported immediately after the first PBM session (T1) and 5 h afterward (T2) and continued to improve at T3 where all the subjects were pain-free. This signifies that employing a lower therapeutic power output is an important pillar for achieving optimal outcomes.

#### 4.1.2. Assessment of Pain Alleviation at Functional Activities

Pain is a major factor that can interfere with patients’ eating, drinking and speaking, which can have a great impact on their quality of life (QoL). 

In the present study, at T0, all the subjects reported the worst possible pain at a mean value of “9.6” on the NPIA during functional activities (speaking, eating, speaking and brushing teeth). However, immediately after the first PBM session (T1), the pain intensity was significantly reduced to a value of “6.5” on the NPIS (moderate pain), but we observed significant pain intensity reduction at rest, where the mean score was “5.66” on the NPIS at T1 (50% pain reduction). This signifies that functional movement can contribute to pain intensity. Nevertheless, in our study, we observed a significant reduction in patients’ pain intensity at T2 (5 h after the first PBM session) where the mean score was “2.6” on the NPIS. This continued to improve at T3 (immediately after the second PBM session) to a mean score value of <0.5 on the NPIS, indicating complete pain relief, and this was maintained to a score value of “0” at T4, T5 and T6. The authors observed that employing a second session of PBM at an interval of 72 h from the first session was a necessity to induce complete pain relief during the functional activities. As the literature has only one study that utilised PBM in treating RAS in the paediatric and adolescent cohort and pain intensity during the functional activities was not assessed [52], a comparative evaluation would not be possible. Nevertheless, there are a few case reports in the scientific literature where PBM was employed in treating RAS in adults.

### 4.2. Appraisal of Wound Healing Rate

PBM laser irradiation showed to be effective in enhancing the wound healing rate and accelerating lesion resolution [43,44,45,49]. 

MiRAS lesions are the most common RAS subtype of a diameter < 5 mm. Despite the lesion frequent tendency of recurrence at a rate of 50% every 3 months [3], it heals within 10–14 days without scarring [74]. Whereas, MaRAS are less common than MiRAS lesions (~10–15% of all RAS). These lesions are similar in appearance to those of minor RAS; however, they are larger (>5 mm in diameter), deeper, result in scarring and can last for weeks or even months [53,75].

There is a difference in the RAS healing rate among children, adolescents and adult. The scientific evidence showed that RAS flared at a higher frequency in children, but the duration of the inflammatory attacks was longer lasting in adults [76]. In this context, all the subjects in our study were followed at different and long-term timepoints: T4, T5 and T6. None of them had a lesion recurrence.

The findings of our study showed a significant reduction in the lesion surface area at T3 compared to T0, indicating >50% complete healing (Grade II) and complete healing and lesion resolution (Grade I, 100%) at T4, apart from case #4, where complete healing was observed at T5, due to a lack of oral hygiene compliance, which is one of the predisposing factors in promoting inflammation. At T5 and T6, none of the subjects had lesion recurrence. The wounds of all the subjects regardless of their RAS subtypes healed without scarring, which is in disagreement with the literature suggesting MaRAS wounds can heal with scarring [67].

In terms of erythema evaluation, all the subjects showed a significant reduction in erythema based on the CEA visual assessment scale at T3 and continued to improve where >50% of the subjects showed no sign of erythema, but case #1 (MaRAS) had a slight redness (almost clear) (Grade 1 on CEA) and case #4 had mild erythema (Grade 2). Nevertheless, all the subjects had no erythema (Grade 0) at T5 and T6. 

The healing rate and complete lesion resolution were faster in our study, taking into account our study’s cohort had different clinical presentations of RAS subtypes, compared to other published studies [28,52,77], in which erythema reduction and ulcer healing improvement were significantly higher in subjects in the PBM group irradiated with λ 810 nm compared to those in the control group by day 3. Another clinical study conducted by Dhopte et al., 2022 [73] showed a reduction in ulcer size in the PBM group compared to the control (pharmacotherapy) on the fifth day of complete treatment.

Employing low therapeutic output power irradiation can stimulate reepithelialisation of the wounds [78] by increasing the metabolism of the mitochondrial respiratory chain which in turn upregulates mitotic activity, collagen synthesis and epithelial proliferation [79].

### 4.3. Patient’ Compliance and Cooperation

Our results showed that PBM is an acceptable therapy in the paediatric and adolescent cohort as all the subjects scored level “4” on the FBRS at T1 and T3. This coincides with the findings of an RCT conducted by Bardellini et al., 2020 [52]. 

### 4.4. PBM Feasibility, Safety and Patient Satisfaction

Several studies have shown that PBM can be considered a reliable, safe and alternative therapy to topical steroids in the management of RAS, since it was more effective in reducing both pain intensity and ulcer size [73,80]. This was supported by a recent systematic review conducted by Khaleel et al., 2020 [50] that included five RCTs comparing PBM versus different pharmacotherapies such as triamcinolone acetonide, granofurin and solcoseryl and others. In all their included studies, the subjects treated with PBM reported lower pain scores and a shorter wound healing time. Moreover, caution needs to be considered when systemic treatment is prescribed to paediatric and adolescent patients and should only be considered in children with compromised immunity [3], and pentoxifylline is one of these treatments. It inhibits TNF-α production and other pro-inflammatory cytokines [1], and hence, it is not indicated in patients who are <18-year-old [29,30]. Corticosteroid medications can cause candidiasis [4], and oral rinses based on tetracycline or chlorhexidine content can shorten the ulcer healing time, but they are contraindicated in children and can induce tooth discoloration in paediatric and adolescent patients [4,31]. 

In line with the abovementioned notes and taking into account the side effects of pharmacotherapy in paediatric and adolescent patients, PBM therapy, in our study, showed to be safe with no side effects and with a great feasibility among our cohort who reported “very good” treatment satisfaction, and this was also reflected by their parents’ positive experiences. 

### 4.5. PBM Dosimetry and Treatment Protocol Evaluation

The effect of PBM therapy on wound healing was examined in in vitro and in vivo experiments [45,70] and showed PBM accelerated wound healing by promoting cell proliferation and procollagen synthesis, accelerating the formation of granulation tissue, increasing ATP synthesis within the mitochondria [45,70] and upregulating cytokines and growth factors [44]. The effects are dependent on irradiation parameters like wavelength, output power and energy density. Two RCTs conducted by Alberkton et al., 2014 (*n* = 20) [77] and Aggarwal et al., 2014 [28] utilised different dosimetry and treatment protocols in treating RAS in an adult cohort as follows, respectively: 809 nm, 60 mW, 80 s, 1800 Hz, beam in contact with tissue and single irradiation for three days [77] and λ 810 nm, 0.5 W, non-contact (2–3 mm) and three irradiations in one day (each lasting was for 45 s with a 30–60 s gap for a total of 3 min) [28]. Both studies reduced pain and enhanced wound healing in treating RAS in the adult cohort. However, none of them mentioned whether a power meter was employed to measure the therapeutic output power reaching the target tissue. Moreover, the beam profile in those studies was Gaussian where the energy is at its maximum in the centre of the targeted lesion surface area but a with a loss of >90% at the periphery [55,56]. Additionally, the methods of randomisation were not described, and no long-term follow-up was reported to ensure no evidence of lesion recurrence. Hence, it would be difficult to extrapolate any reproducible PBM laser dosimetry and treatment protocol from those studies. 

The only RCT study up to date that utilised a PBM laser in the management of MiRAS in a cohort aged between 5 and 12-years-old was conducted by Bardellini et al., 2020. They did not investigate the use of PBM therapy in other RAS clinical presentations. They utilised the following parameters and treatment protocol: λ 645 nm, 100 mW, 1 cm^2^, CW, a fluence of 10 J/cm^2^ and three consecutive days of irradiation in a CW, but the irradiation time was not reported. The randomisation protocol was reported. The follow-up timepoint was up to 10 days. Knowing the high recurrence rate of RAS among the paediatric and adolescent cohort (50%) at 3 months [3], it is essential to obtain a long-term follow-up to ensure the sustainability of the PBM therapy. Hence, in our study, we followed up the subjects three days after the complete PBM therapy (T4) and at two-week (T5) and three-month (T6) timepoints. Moreover, using λ 645 nm, which is a red light, offers a shallow penetration, and hence employing a lower power of 100 mW (no power meter mentioned to measure the therapeutic power reaching the target) can compromise the clinical outcomes. Therefore, in our study, we utilised λ 980 nm laser PBM which offers a deep penetration depth [54] with the therapeutic output power measured with a power meter at a value of 300 mW delivered with a flattop beam profile hand-piece which irradiated the lesion for 60 s, and the protocol was repeated at 72 h time intervals, ultimately leading to the optimisation of the clinical outcomes, taking into account the RAS subtypes.

### 4.6. Study’s Limitation and Future Perspectives

The limitations of the study are as follows: (1) lack of a controlled longitudinal observational study on a series of subjects receiving the same intervention (i.e., there was no control/sham group); (2) a low number of treated patients; (3) level IV evidence-based medicine; (4) little statistical validity due to a lack of the control group to compare the outcomes; and (5) a lack of a quantitative assessment tool. 

Despite the abovementioned study limitations, our PBM laser dosimetry and treatment protocols addressed all the setbacks of the published RCTs in the scientific literature [28,52,73,77] and was superior to provide immediate pain relief at T1 and T2, rapid wound healing at T3 and complete lesion resolution at T4 compared to the abovementioned paediatric RCT [52] and RCTs for the adult cohort. [28,73,77]. This signifies that PBM dosimetry and treatment protocols in the present study were not only robust and offered optimal clinical outcomes but also proved PBM’s sustainability at a long-term follow-up with no evidence of recurrence. This ultimately can be reproducible in future extensive studies. Hence, long-term, randomised, controlled and large sample-sized clinical trials should be conducted to validate the dosimetry and treatment protocols.

Moreover, PBM therapy is constantly evolving and it is changing the way we work, allowing us to achieve optimal clinical outcomes, improve patients’ experience, especially in paediatric and adolescent patients, and also offer scientific work reproducibility as clearly demonstrated in our study. It is noteworthy that PBM therapy signifies not only efficacy in treating RAS in paediatric and adolescent patients with optimal clinical outcomes, safety and feasibility, but it also reduces CO_2_ consumption, offers cost-effectiveness in the long term [81] and enhances sustainability.

## 5. Conclusions

This is the first report in the scientific literature that demonstrates the efficacy of λ 980 nm in the management of all RAS subtype in paediatric and adolescent patients based on a 3-month follow-up period, and the results showed a promising λ 980 nm laser as an immunomodulatory therapy in not only reducing the healing time and offering complete lesion resolution but also exerting immediate pain relief with no evidence of recurrence in long-term follow-ups. Hence, we reject our null hypothesis.

Our study’s dosimetry and treatment protocols were effective from scientific and practical standpoints, and hence, extensive studies are warranted to validate its reproducibility. The authors recommend multicentre RCTs with larger samples in order to enrich our knowledge in PBM application in all RAS subtypes. 

## Figures and Tables

**Table 1:** Photobiomodulation (PBM) laser device specifications, PBM parameters and treatment protocol.

Device specifications	Manufacturer	Doctor Smile, Lambda, Italy
	Model identifier	Wiser 2
	Emitters type	Diode laser
	Medical/laser class	IV
	Beam delivery system	Fibre
	Probe design	Single
	Beam profile	Flattop
	Beam divergence	0°
Irradiation parameters	Wavelength (nm)	980
	Therapeutic power output (W)	0.3
	Emission mode	CW
	Beam spot size at target (cm^2^)	1
	Irradiance at target (W/cm^2^)	0.3
	Energy per spot (J)	18
	Fluence (J/cm^2^) per point	18
	Irradiation time (s) per point	60
Treatment protocol	Number of irradiated point per case	1
	Laser–tissue distance (mm)	2 (non-contact)
	Application technique	Static
	Total treatment sessions per week	2
	Frequency of session/week	Twice a week
	Time interval	3 days (72 h)

**Table 2:** Clinician erythema assessment (CEA) visual assessment scale [59].

Grade	Description of the Erythema
0	Clear; no sign of erythema
1	Almost clear; slight redness
2	Mild erythema; definite redness
3	Moderate erythema; marked redness
4	Severe erythema; fiery redness

**Table 3:** Subjects’ demographic characteristics. Abbreviations: F: female; M: male; UR3: upper right canine; UR4: upper right first premolar; LL1: lower left central incisor; URc: upper right deciduous canine; LR1: lower right central incisor; yrs: year; h: hour.

Case #	Gender	Age (yrs)	Lesion Onset (h)	RAS Type	Lesion Site	No. of Lesions
1	M	11	~6	Major: 1 Herpetiform: 7	Keratinised and non-keratinised buccal mucosa of UR3 and UR4, extending to buccal vestibule	8
2	M	13	~10	Minor	Keratinised labial mucosa of LL1	1
3	F	12	~10	Minor	Non-keratinised buccal mucosa of inner cheek, opposite to LR4	1
4	F	6	~12	Major/Minor	Keratinised and non-keratinised buccal mucosa of URc, extending to the buccal sulcus	2
5	M	7	~10	Minor	Keratinised buccal mucosa of URc	1
6	F	7	~24	Major	Non-keratinised labial mucosa of LR1, extending to the labial sulcus and lower midline frenum	1

**Table 4:** The type of the RAS, number of lesions for each subject including the wound surface area progression at T3, T4, T5 and T6 compared to T0, as well a complete resolution and any evidence of lesion recurrence. Abbreviations: CC: complete closure; NR: no lesion recurrence; RAS: recurrent aphthous stomatitis; MiRAS: minor recurrent aphthous stomatitis; MaRAS: major recurrent aphthous stomatitis; T0: pre-treatment; T3: second PBM session (three days post first PBM session); T4: three-day follow-up (after complete PBM treatments); T5: two-week follow-up; T6: three-month follow-up.

Case #	RAS Type	No. Lesion	Wound Surface Area Progression Measured in mm^2^; CC and NR				
			T0	T3	T4	T5	T6
1	MaRAS	1	5.6	2	CC	CC	NR
	herpetiform	7	0.6–3.3	6 lesions = 0; 1 lesion = 0.5			NR
2	MiRAS	1	2.8	2.5	CC	CC	NR
3	MiRAS	1	3.4	1.5	CC	CC	NR
4	MiRAS	1	4.5	1	CC	CC	NR
	MaRAS	1	8	2	0.5		
5	MiRAS	1	4.5	2	CC	CC	NR
6	MaRAS	1	15	CC	CC	CC	NR

## Data Availability

All data are included in the text.
